# Challenges for the diagnosis and treatment of malaria in low transmission settings in San Lorenzo, Esmeraldas, Ecuador

**DOI:** 10.1186/s12936-018-2591-z

**Published:** 2018-11-28

**Authors:** Mayte Mosquera-Romero, Lina Zuluaga-Idárraga, Alberto Tobón-Castaño

**Affiliations:** 1 National Faculty of Public Health, University of Antioquia, Medellin, Colombia; 2 Malaria Group, Faculty of Medicine, University of Antioquia, Medellín, Colombia

**Keywords:** Malaria, Barriers, Diagnosis, Therapeutics, Implementation research

## Abstract

**Background:**

Ecuador is on the verge of eliminating malaria according to the World Health Organization criteria. Nevertheless, active transmission foci still persist in the country, and these represent an important challenge for achieving the objectives set out. Diagnosis and treatment are a mainstay in the control and elimination of this disease. This study aimed to explore the barriers hindering the implementation of malaria diagnosis and treatment strategies in a focus of active transmission in the San Lorenzo canton, Ecuador.

**Methods:**

Using a convergent mixed methods design during 2017, the researchers assessed the physical and human resources of the services network at the primary level of care along with the quality assurance activities, patient access to healthcare services and perceptions regarding the care provided to patients with malaria.

**Results:**

The programme’s administrative transition from the National Service of Vector-borne Diseases to the Ministry of Public Health is perceived from the interviewed participants to have weakened the diagnosis network established in recent years. A mean of 6.4 ± 0.88 months was found for anti-malarial medication shortage at the primary level of care. Likewise, there was high healthcare staff turnover (permanence, Me = 7 months; IQR = 5–16) and a deficit of general knowledge on the disease among the entirety of healthcare staff, as only 29% of physicians were aware of the correct first-line treatment for malaria by *Plasmodium falciparum* and *Plasmodium vivax*. It was evidenced that 95.7% of patients were hospitalized to receive anti-malarial treatment. Both patients and healthcare staff considered the area to be difficult to reach due to its geography and the presence of groups outside the law. They also identified the lack of personnel and microscopy posts in this border area as the main barrier.

**Conclusion:**

The network of diagnostic services for malaria is weak in San Lorenzo, and socio-economic, political and historical factors hinder the implementation of the universal malaria elimination strategy based on diagnosis and treatment.

## Background

Malaria still represents a major public health issue despite the important reductions in the mortality and morbidity rates of malaria achieved worldwide by the implementation of several anti-malarial strategies. It is expected that all endemic countries eliminate malaria by adapting the available interventions to their local context and combining them [1]. Therefore the Global Technical Strategy for malaria 2016–2030 is based on five principles, the first of which is universal access to malaria prevention, diagnosis and treatment [2].

Ecuador has reduced malaria incidence in more than 90% [3]. This resulted in low and very low transmission settings, leading the country towards possible elimination of the disease as per requirements of the World Health Organization (WHO) [4]. The implemented strategies have followed the recommendations of WHO, prioritizing diagnosis, treatment, vector control and epidemiological and entomological surveillance [3]. The National Service of Arthropod Vector-Borne Diseases SNEM (initials in Spanish) was previously in charge of such strategies. However, in 2014 a political transition process transferred its functions and competences to the National Directorate of Prevention and Control (NDPC), which belongs to the Public Health Ministry (PHM) [5].

In spite of the achievements in the battle against this disease, Ecuador still has active transmission foci whose main characteristic is that malaria incidence has not decreased compared with the other regions of the country. Such is the case of San Lorenzo canton, whose population, healthcare services and eco-epidemiological factors have particular conditions that render the reduction of malaria cases uncertain [6].

Studies conducted in countries which have succeeded in eradicating malaria identify reduced political and financial commitment as the main threat to the success of anti-malarial strategies, along with weak healthcare service infrastructures [7]. To approach such a complex issue in Ecuador, a study was conducted including qualitative and quantitative methods in a mixed approach to evidence the barriers existing in terms of service, healthcare staff and patient organization, as well as in the implementation of diagnosis and treatment strategies. This study included an assessment of the physical and human resources, quality, service accessibility, and perception of the patients with malaria in the particular context of the San Lorenzo canton.

## Methods

### Design and studied population

This study aimed to explore the barriers hindering the implementation of malaria diagnosis and treatment strategies in an endemic area for malaria transmission in the San Lorenzo canton (Esmeraldas, Ecuador) during 2017. A convergent mixed method was designed [8]. Three questionnaires were applied for the quantitative assessment to record data obtained from observation and verification of human and physical resources that healthcare institutions had for malaria diagnosis and treatment. Healthcare staff’s general knowledge regarding malaria and patients’ access to healthcare services were also assessed.

The study included all healthcare institutions at the primary level of care of the PHM (14 in total). All personnel from each unit received an invitation to participate in the study by making an appointment. Patients diagnosed by the PHM during the first semester of 2017 were invited to participate in the accessibility survey. These individuals were identified using the database provided by the surveillance team of the District Health Office of San Lorenzo.

The qualitative assessment was conducted via semi-structured interviews with staff in charge of the malaria programme at the three administrative levels of the PHM (central, zonal, district), the patients and the healthcare professionals to inquire about the perception of the healthcare services via discourse analysis.

### Statistical analysis

Quantitative data was analysed using the Stata 14 statistical software (StataCorp LP, TX, USA). The quantitative variables are presented using descriptive statistics (mean and standard deviation or median and interquartile ranges) depending on their normal distribution, evaluated through the Shapiro–Wilk test. Qualitative variables were described using absolute frequencies and proportions in percentage. Interviews were transcribed using Microsoft Word and analysed using the Atlas Ti software (both licensed to the University of Antioquia).

### Ethical aspects

This study was approved by the Ethical Review Board of the National Faculty of Public Health of the University of Antioquia, in Colombia (Act 156, 6 December 2016). In Ecuador, it was approved by the Ethical Review Board for Research on Human Beings of the Pontifical Catholic University of Ecuador (Letter CEISH-264-2017) and the Health Intelligence Directorate of the Ecuador Ministry of Public Health (Letter MSP-DIS-2017-0057-O), with permission from the respective district of the San Lorenzo canton. All participants signed an informed consent form.

## Results

### Organizational dynamics and structure of the diagnosis network

The service network of the PHM in the San Lorenzo canton is composed of 14 primary-level healthcare institutions and one secondary-level hospital. The researchers visited all the primary-level centres, as the point of entry to the health system. Service organization was assessed by applying three interviewers and a questionnaire to each operative unit.

The responses of the interviewed participants showed that the PHM has made an effort to prioritize care for this disease at every operative level in order to achieve the goal of eliminating malaria from Ecuador. Nevertheless, several challenges arise which prevent full implementation of the strategies.

The integration of the SNEM into the PHM was perceived by the interviewed participants as a shortcoming, since they considered that malaria was a priority when an institution focused exclusively on it. Moreover, the transition could not be carried out entirely, as expressed by an officer from the PHM:

*“We are aware of the fact that we still have ways to go. Like I said before, the paperwork is physically ready, but we still haven’t truly reached the territories” (AD-06)*.

Furthermore, the personnel from the PHM stated that, at the operative level, they did not have the updated normative documentation on malaria because it was being written or reviewed by the competent directorates.

Each healthcare institution had at least one physician, a nurse, an obstetrician or a dentist working 8 h a day, and one institution had a 24-h emergency unit. Since they were located in scattered areas and San Lorenzo is a border canton, in most cases the medical staff had consecutive working days assigned monthly and while on days off or on vacation there was no available replacements, leaving some areas without physicians for 7–15 days. The healthcare staff were trained in sample collection for thick blood smear and rapid diagnostic tests (RDTs) across the entire network of services, regardless of their professional role. The entire Health District had four microscopists, a situation perceived as a deficiency of the diagnosis network, particularly in the border area.

*“We have four microscopists in the canton. However, that amount is not enough to reach the malaria elimination criteria, and we are developing proposals to increase the staff in at least three additional areas”* (AD-05).

It was confirmed through observation that all healthcare institutions had the necessary elements for diagnosing malaria, both in terms of microscopy sample collection and RDTs. Anti-malarial medication was available for *Plasmodium falciparum* in one of the 14 primary-level institutions. In contrast, no medication was found for *Plasmodium vivax* in any of the institutions. According to the participants, there was a drugs shortage in the past 6.43 ± 0.88 months, on average. The staff explained that the shortage lied in the strategy used in the canton because every *P. falciparum* malaria cases in the previous 3 years had been hospitalized, and the priority had been keeping the medication in the secondary-level hospital.

*“In the past, it [anti-malarial medication] was given to all operative units when there was enough in stock. But in the last months, the demand in this hospital has increased and, according to statistical data, more than 95% of our patients accept hospitalization, thus having the medication where it is most required has become the priority (…)”* (AD-07).

The interviewees acknowledged the importance of strengthening the diagnosis network at the border, where a high number of people cross over. They also highlighted the lack of microscopic diagnosis posts in these key areas as one of the main barriers preventing Ecuador from achieving its goal of malaria elimination.

*“One of the first barriers we encountered is the lack of well equipped microscopic diagnosis posts in key places such as the border, where there is currently a lot of people crossing, particularly Colombians and Venezuelans”* (AD-06).

### Healthcare professionals for malaria patients

A total of 45 healthcare professionals from the primary-level institutions were included. Female represented 69% of these and 87% worked in rural areas. The median age was 31 years (IQR 27–32) and the median for permanence at the working places was 7 months (IQR 5–16). Most participants were physicians (31%), followed by nursing graduates (24%) (see Table 1).

**Table 1:** Characterization of the healthcare staff working at the primary-level institutions of the PHM included in this study

Features	No. of participants n = 45; n (%)
Sex	
Male	14 (31.1)
Female	31 (68.9)
Operating unit area type	
Rural	39 (86.7)
Urban	6 (13.3)
Type of healthcare professional	
Physician	14 (31.1)
Nursing graduate	11 (24.4)
Nursing assistant	1 (2.2)
Obstetrician	8 (17.8)
Dentist	7 (15.6)
Microscopist	4 (8.9)
Workload (h)	
6	5 (11.1)
8	40 (88.9)
Function	
Blood sampling	45 (100.0)
Clinical diagnosis	14 (31.1)
Microscopic diagnosis	4 (88.9)
Diagnosis using RDTs	45 (100.0)
Treatment	9 (20.0)
Quality assurance	
Supervision (last 6 months)	25 (51.1)
Training (last year)	29 (64.4)

### Knowledge about the disease

All participant stated they had been trained for preparing thick blood smear tests and for performing diagnoses with RDTs. They also expressed this was part of their functions. Additionally, 64% of them reported having been trained during the previous year, and 51% was supervised by higher-rank institutions at their work stations during the last 6 months (Table 1).

The knowledge survey revealed that 91% of the interviewed staff suspected the presence of malaria upon encountering febrile patients during consultation, while 7% recognized the classic triad of chills, fever and sweating. Finally, 86% of physicians (n = 12) stated they knew at least thick blood smear test and RDTs as diagnostic tests (Table 2).

**Table 2:** General knowledge on malaria as observed among the healthcare professionals working for the healthcare institutions at the primary level of care of the PHM

	Physicians n = 14; n (%)	Other staff n = 31; n (%)	Total n = 45; n (%)
Suspected disease upon encountering a febrile patient			
Malaria	11 (78.6)	30 (96.8)	41 (91.1)
Dengue	11 (78.6)	15 (48.4)	26 (57.8)
Chikungunya	9 (64.3)	9 (29.0)	18 (40.0)
Zika	9 (64.3)	8 (25.8)	17 (37.8)
Other	4 (28.6)	6 (19.4)	10 (22.2)
Methods for diagnosis			
Thick blood smear and RDTs	12 (85.7)	29 (93.6)	41 (91.1)
Malaria-related symptoms			
Classic triad	0 (0.0)	3 (9.7)	3 (6.7)
Fever	14 (100.0)	30 (96.8)	44 (97.8)
Cephalea	6 (42.9)	18 (58.1)	24 (53.3)
Diaphoresis	2 (14.3)	4 (12.9)	6 (13.3)
Chills	1 (7.1)	11 (35.5)	12 (26.7)
Myalgia	5 (35.7)	8 (25.8)	13 (29.0)
Arthralgia	6 (42.9)	8 (25.8)	14 (31.1)
General exanthema	0 (0.0)	4 (12.9)	4 (8.9)
General discomfort	4 (28.6)	9 (29.0)	13 (28.9)
Abdominal pain	2 (14.3)	1 (3.2)	3 (6.7)
Pruritus	0 (0.0)	1 (3.2)	1 (2.2)
Conjunctivitis	0 (0.0)	1 (3.2)	1 (2.2)
Vomiting	1 (7.1)	3 (9.7)	4 (8.9)
Retro-ocular pain	3 (21.4)	3 (9.7)	6 (13.3)
Severe symptoms			
Persistent fever	3 (21.4)	6 (19.4)	9 (20.0)
Persistent vomiting	0 (0.0)	3 (9.7)	3 (6.7)
Altered consciousness	2 (14.3)	3 (9.7)	5 (11.1)
Seizures	1 (7.1)	0 (0.0)	1 (2.2)
Jaundice	3 (21.4)	2 (6.5)	5 (11.1)
Haemorrhage	4 (28.6)	3 (9.7)	7 (15.6)
Signs of dehydration	5 (35.7)	6 (19.4)	11 (24.4)
Hyperparasitaemia	0 (0.0)	0 (0.0)	0 (0.0)
Causal agent			
*Plasmodium*	11 (78.6)	9 (29.0)	20 (44.4)
Transmission mechanism			
Mosquito bite	14 (100.0)	30 (96.8)	44 (97.8)
Transmitting vector			
*Anopheles*	7 (50.0)	11 (35.5)	18 (40.0)
Plasmodium that infects humans			
At least 2 species	12 (85.7)	21 (67.7)	33 (73.3)
At least 3 species	6 (42.9)	4 (12.9)	10 (22.2)
Plasmodium species circulating in the country			
*P. vivax* and *P. falciparum*	9 (64.3)	17 (54.8)	26 (57.6)
Prevention and control measures			
Repellent	10 (71.4)	24 (77.4)	34 (75.6)
Adequate clothing	3 (21.4)	9 (29.0)	12 (26.7)
Mosquito net	12 (85.7)	24 (77.4)	36 (80.0)
Fumigation	4 (28.6)	1 (3.2)	5 (11.1)
Intra-domiciliary spraying	0 (0.0)	0 (0.0)	0 (0.0)
Controlling breeding sites	11 (78.6)	26 (83.9)	37 (82.2)

Regarding the transmission mechanisms of the disease, 98% of the healthcare staff identified mosquito bites and 40% the *Anopheles* vector. As for the species of circulating agents in the country, 58% of the participants answered correctly by mentioning the presence of *P. vivax* and *P. falciparum* (Table 2).

In addition, 29% of physicians (4/14) knew the first-line treatment for *P. vivax* and *P. falciparum* as per national guidelines. Globally, less than 10% of the participants were correct in their answers regarding first-line treatments for severe malaria and malaria during pregnancy (Table 3).

**Table 3:** Knowledge of the first-line treatment for malaria among the healthcare staff

Correct first-line treatment	Physicians n = 14; n (%)	Other staff n = 31; n (%)	Total n = 45; n (%)
*P. vivax* ^a^	4 (28.6)	7 (22.6)	11 (24.4)
*P. falciparum* ^b^	4 (28.6)	5 (16.1)	9 (20.0)
*P. vivax* among pregnant women^c^	1 (7.1)	3 (9.7)	4 (8.9)
*P. falciparum* during the first trimester of pregnancy^d^	1 (7.1)	1 (3.2)	2 (4.4)
*P. falciparum* during the second and third trimesters of pregnancy^e^	0 (0.0)	2 (6.5)	2 (4.4)
Treatment for severe malaria^f^	0 (0.0)	2 (6.5)	2 (4.4)

^a^Chloroquine–primaquine

^b^Artemether–lumefantrine and primaquine

^c^Chloroquine

^d^Quinine–clindamycin

^e^Artemether–lumefantrine

^f^Artesunate

The National Institute of Public Health Research (NIPHR) is the executing and governing entity of the quality assurance for microscopic diagnosis (QAMD). The Programme for external performance evaluation (PEPE) is among its activities, including a theoretical component consisting of a pre-test and a post-test graded as percentages of correct answers. During this study the results of the PEPE were collected from the four microscopists evaluated. The results were satisfying for all the microscopists of the studied canton, with concordance rates of 90% for positivity and species.

### Patients with malaria treated by the PHM network

During the first semester of 2017, a total of 99 *P. falciparum* malaria cases were reported in the surveillance system of the health district. There were 54 patients located by the researchers who agreed to participate in the study. A total of 31 individuals (57%) were male, with a median age of 17 years (IQR 11–26), and 52% self-identified their ethnicity as “Afro-Ecuadorian” or black. In addition, 91% of the patients were Ecuadorian and 13% were affiliated to the health regime of the Ecuadorian Institute of Social Security (EISS). Approximately 57% of the patients had completed at least elementary school, and the most common occupations were: student, palm grower, housewife and miner.

Access to healthcare services for the patients of the San Lorenzo canton is influenced by their own characteristics and residence locations. Their narrative regarding the attention process showed that they have to overcome inherent difficulties to reach healthcare institutions to be cared by medical staff during the established schedule. The entire healthcare staff has been instructed to perform thick blood smear tests and apply RDTs to diagnose malaria. Depending on the first available result, once a case of malaria has been identified, the diagnosis is explained to the patient and inpatient treatment is then offered to *P. falciparum* cases at the secondary-level institution, according to the national policy.

This study found that most patients agreed to receive inpatient treatment. There were 51 cases (94%) receiving this type of care with full compliance of the therapeutic scheme as per national guidelines. The median duration for hospitalizations was 4 days (IQR 3.5–4) with at least three follow-up controls to check for negative parasitaemia in 30% of patients.

According to the survey, the patients who had a job had an average weekly income of US$70 ± 35.08 obtained from activities mainly related to agriculture, fishing and mining. During the interviews they discussed the informality of their jobs, the delays in their payments and how limited their income is to support their families.

It was confirmed that, due to the national policy of free public healthcare, patients do not incur any direct healthcare-related fees. However, there are other expenses related to transportation to primary healthcare facilities and later to hospital. Furthermore, there is an opportunity cost resulting from hospitalization. Many patients do not have enough money to pay for transportation, as described by one of the healthcare professionals:

*“The patients themselves say ‘I have no money’, and this is where we try to help. I have approached the social work office directly and, to tell you the truth, it’s very bureaucratic (…) the answer is that they are going to visit the hospital and, once they do that, they’ll see whether it’s necessary to help them or not, and nothing is for sure”* (LO-08).

Likewise, during the interviews patients expressed their opinions regarding transportation costs. These are dependent on the particular location from which they need to travel. These values range from 1.00 to 25.00 dollars.

*“If it is during the night, it’s necessary to rent a car or make a call. It’s difficult. It’s expensive for us because we are poor. Around US$25. It’s difficult to get that much money to go to the healthcare centre”* (CO-03).

The interviews and visits across the canton showed that some areas are difficult to access, either due to limitations in the means of transportation, road conditions or areas controlled by informal mining actors and groups outside the law.

*“The difficulty lies in the fact that this population, because of the geography of San Lorenzo, has many variations: there are communities that can only be accessed from the river, (…) controlled by illegal miners and guerrilla groups (…) a critical and very important point for the correct monitoring of the population in order to prevent outbreak expansion”* (AD-05).

The patients stated that they move around the canton mostly by taxi (69%) because of irregular schedules and dependance on passenger demand of other means of public transport. Such is the particular case of the La Cadena parish, where, trucks with double transmission are required to access these hard-to-reach locations.

Different times could be established from the start of the search for healthcare. The median time for a journey from a patient´s residence to the healthcare facility was 30 min (IQR 10–60) based on the usual possibilities of transportation. The median time between the start of consultation and the delivery of diagnosis was 60 min (IQR 30–180). Finally, the median time from positive diagnosis to the reception of the first dose of treatment was 240 min (IQR 120–720) Based on the operational definition of geographic access to healthcare service in less than an hour employing the usual means, it was found that 9% of the patients encountered geographical barriers. The results also showed that only 22% of patients received prompt treatment as per WHO recommendations of providing anti-malarial treatment within 24 h of onset to every patient with malaria symptoms [9].

Although there are ethnical minorities in the canton who speak a language—other than Spanish, the results did not show this to be a barrier hindering communication. Even during the interview with a member of the Awá community, he explained that Spanish is an extensively known language in their communities.

Of the surveyed patients, 52% stated that they had requested consultation immediately after suspecting the presence of the disease, while 54% said they initially went to the healthcare institutions of the PHM. Other behaviour mentioned by patients included: self-medication (32%), asking relatives or neighbours (9%), consultation with the private sector (4%), and using traditional medicine (2%).

*“When it happens suddenly, we go to the traditional doctor, otherwise I cure it myself (…), if we don’t get better, we then go to the healthcare centre”* (CO-02).

Approximately half of patients did not usually visit healthcare facilities when they felt sick. This decision is probably influenced by the perceptions of the PHM services in the canton. The participants also expressed that they did not trust the staff from the healthcare centre, either because of their lack of attention during consultation or because they felt mistreated at the institution.

*“(…) One day some doctors came; nowadays they don’t even examine you: they only ask where it’s hurting, what you feel and that’s it. They then give you some little pills and some painkiller (…).”* (CO-02).

There are additional individual conditions preventing patients from accepting healthcare or complying with follow-up controls, e.g., participating in illegal activities. In consequence, the staff from the PHM acknowledged the difficulty of working with this population.

Despite this, the community still considers malaria as a major health problem and tries, within its possibilities, to prevent it. This is why 93% of the participants use mosquito nets, and 83% said theirs was in optimal conditions.

The staff from the PHM believed that continuous exposure of the population to the disease has not necessarily had a positive influence; on the contrary, it has caused the disease to be underestimated, leading to a lack of awareness of the real risk. This is another important challenge.

*“As a barrier, I would include the population’s lack of education and awareness. Although this topic is frequently discussed, since there are no statistics about people dying from it, people do not understand the seriousness of the matter and the disease is underestimated. This could be the first complication.”* (AD-07).

## Discussion

Diagnosis and treatment strategy is one of the fundamental pillars to achieve the goal of eliminating malaria. Its importance is expressed in all the guidelines and recommendations of the WHO [2, 9]. Ecuador intends to eliminate this disease by implementing the Strategy and Action Plan against Malaria, which contains goals and objectives aligned with the Global Technical Strategy for malaria 2016–2030 [2, 10].

This study managed to identify barriers hindering the implementation of malaria diagnosis and treatment strategies in terms of service organization, healthcare professionals and patients. This makes it imperative to have an intervention aligned with the malaria elimination goals in Ecuador.

One of the greatest challenges faced by countries in the process of eliminating malaria is related to political instability, which reduces compliance and commitment to anti-malarial interventions and causes a decrease in financial resource allocation [7]. It is possible to perceive from the healthcare staff how the transition from the SNEM to the PHM did not include a complete transfer of information, technologies and competences, which probably contribute to weaken the diagnosis network, along with programme instability. Moreover, there is a lack of official documents regarding malaria patient care at local levels.

The decrease in the total incidence of malaria cases during the last decade places Ecuador in a possible elimination scenario. It is imperative to consolidate programmes to maintain the already achieved goals. In a study by Ferreira et al. [11] conducted in Brazil, it was observed that tendencies towards a decrease might cause the disease to be underestimated and displaced by other health-related priorities emerging in the country. This makes it important to permanently assess programmes to achieve and maintain malaria elimination, and to have technical, operational and financial feasibility which makes it possible to value the viability of the goals established [12].

The service network of the PHM has the physical resources required to diagnose malaria via microscopy and RDTs in the canton and the entire primary level of care. However, there are limitations regarding the supply of anti-malarial medication. This is an important barrier hindering timely provision of treatment.

Additionally, there are deficiencies in human resources. Based on these limitations, the Health District has organized services to improve their availability to patients by training all healthcare personnel in activities related to malaria diagnosis. However, there is no full coverage or collaboration between different official and private sectors (e.g. education, agriculture, environment). The experience in Laos suggests extending diagnosis and treatment coverage to all febrile patients in endemic areas while leveraging support from the private sector [13]. Although this study did not assess asymptomatic patients or submicroscopic malaria, they are acknowledged as a challenge and a critical reservoir that is sustaining malaria transmission, as pointed out by Stresman et al. [14].

Another barrier identified in the present study is the deficit of general knowledge about malaria observed in all healthcare staff regarding the basic components of the transmission mechanisms, vectors, *Plasmodium* species in Ecuador, clinical manifestations, diagnosis, and treatment. This could be contributing to the rate of misdiagnoses and delays in timely treatment, as demonstrated in other context [15, 16].

Ecuador has a policy of free public healthcare established in the Constitution of the Republic in 2008 [17]; however, patients have additional expenses derived from transportation when seeking healthcare. Such expenses might exceed their payment capacity when they live in remote areas and in poverty, as is the case for malaria patients in this region. The opportunity cost for patients with falciparum malaria, who are hospitalized for 3–4 days to receive treatment should be considered. In Kenya a study shows the relationship between people’s income, transportation expenses and the cost of healthcare services were barriers to prompt treatment [18].

In addition to difficulties related to public order, the canton also has geographical variants hindering the search for medical attention. The present study showed that 48% of patients did not seek medical attention immediately after suspecting the presence of malaria. This could be due to the lack of trust in the healthcare professionals belonging to the healthcare services offered in San Lorenzo. A study in the Bagó Region established a relationship with the higher value perceived in continuing with one’s daily work activities and generating income to support one’s family instead of seeking to relieve symptoms [19]. These situations evidence the relationship between malaria and poverty, especially affecting the most vulnerable populations that benefit only partially from available interventions [20, 21].

Neighbouring countries such as Colombia and Peru have historically had high rates of malaria, making its elimination truly a challenge. This makes cross-border collaboration a requirement given the high rate of national and bi-national migration. This is consistent with the situation experienced in Africa regarding the control and elimination of this disease [22]. The continuous movement of people across borders is related to the temporary nature of their jobs, the emergence of drug resistance and inadequate treatments [23, 24]. These populations should be prioritized in the elimination context, and audacious investing in the social sector would be transcendental for improving the provision of healthcare services and the promotion of cross-border initiatives [25]. As well as compliance with an extension of the terms of currently valid bi-national agreements with Colombia [26].

The elimination activities and goals should be socialized within the communities in order to empower them and increase sensibilization and education so that the strategies can be implemented correctly. In Malawi, Dembo et al. [27], observed that the beliefs and traditions of the people act as barriers and become challenges for the implementation of interventions against malaria. Moreover, it has been proven that anti-malarial interventions are cost-effective in the mitigation of poverty, creating a healthier, more prosperous and equitable society, and that investing in these interventions is justified despite the high costs incurred in order to achieve the goals set [28].

This is the first study conducted in an area of Ecuador where malaria is endemic aiming to identify barriers hindering the implementation of diagnosis and treatment strategies. However, it has several limitations related to data collection from secondary sources provided by healthcare staff, difficulty in reviewing files of patients included in the study, incomplete epidemiological research records, absence in the territory of quality assurance reports for the diagnoses performed by microscopists, together with the geographical access and public order barriers restricting the arrival of all the patients at the healthcare institutions.

Besides, there is an inherent selection bias, since patients who did not access the healthcare service were excluded from the beginning and those who were notified and treated by the PHM network were the ones studied. The biases for the qualitative part are related to the convenience sampling technique used for selecting the key actors of the malaria programme in order to carry out the semi-structured interviews. Moreover, they were aware of the research beforehand, which could have influenced their responses and the preparation of the healthcare institutions before the visit during field work.

## Conclusions

The service network of the PHM is weak in the San Lorenzo canton. This, together with socioeconomic, political and historical factors hinders the universal implementation of strategies for malaria diagnosis and treatment for the country’s malaria elimination goals.

The decrease in the incidence of malaria in Ecuador provides an optimistic outlook hinting to a possible elimination. It is imperative to analyse possible scenarios which include all the identified barriers while ensuring political commitment and stable allocation of financial resources for these activities as a priority.

In this context, the main barriers identified relate to the ability of the PHM service network to provide timely and quality care, the knowledge deficit of healthcare staff working at primary level of care and the barriers hindering patient access to it. As a result, it is urgent to create policies promoting access to healthcare and improving the financial situation of these populations in vulnerable areas. Such policies should have a clear vision and the effective leadership of the national malaria programmes to address the limitations identified in this study, contributing to the control of malaria as an important cause of morbidity in the canton.

## Abbreviations

EISS: Ecuadorian Institute of Social Security
NDPC: National Directorion of Prevention and Control
SNEM: National Service of Arthropod Vector-Borne Diseases
PEPE: program for external performance evaluation
PHM: Public Health Ministry
QAMD: quality assurance for microscopic diagnosis
RDTs: rapid diagnostic tests
WHO: World Health Organization
